# Hyperspectral Imaging With Machine Learning to Differentiate Cultivars, Growth Stages, Flowers, and Leaves of Industrial Hemp (*Cannabis sativa* L.)

**DOI:** 10.3389/fpls.2021.810113

**Published:** 2022-02-02

**Authors:** Yuzhen Lu, Sierra Young, Eric Linder, Brian Whipker, David Suchoff

**Affiliations:** ^1^Department of Agricultural and Biological Engineering, Mississippi State University, Starkville, MS, United States; ^2^Department of Biological and Agricultural Engineering, North Carolina State University, Raleigh, NC, United States; ^3^Department of Crop and Soil Sciences, North Carolina State University, Raleigh, NC, United States; ^4^Department of Horticultural Science, North Carolina State University, Raleigh, NC, United States

**Keywords:** industrial hemp, classification, hyperspectral imaging, image processing, machine learning

## Abstract

As an emerging cash crop, industrial hemp (*Cannabis sativa* L.) grown for cannabidiol (CBD) has spurred a surge of interest in the United States. Cultivar selection and harvest timing are important to produce CBD hemp profitably and avoid economic loss resulting from the tetrahydrocannabinol (THC) concentration in the crop exceeding regulatory limits. Hence there is a need for differentiating CBD hemp cultivars and growth stages to aid in cultivar and genotype selection and optimization of harvest timing. Current methods that rely on visual assessment of plant phenotypes and chemical procedures are limited because of its subjective and destructive nature. In this study, hyperspectral imaging was proposed as a novel, objective, and non-destructive method for differentiating hemp cultivars, growth stages as well as plant organs (leaves and flowers). Five cultivars of CBD hemp were grown greenhouse conditions and leaves and flowers were sampled at five growth stages 2–10 weeks in 2-week intervals after flower initiation and scanned by a benchtop hyperspectral imaging system in the spectral range of 400–1000 nm. The acquired images were subjected to image processing procedures to extract the spectra of hemp samples. The spectral profiles and scatter plots of principal component analysis of the spectral data revealed a certain degree of separation between hemp cultivars, growth stages, and plant organs. Machine learning based on regularized linear discriminant analysis achieved the accuracy of up to 99.6% in differentiating the five hemp cultivars. Plant organ and growth stage need to be factored into model development for hemp cultivar classification. The classification models achieved 100% accuracy in differentiating the five growth stages and two plant organs. This study demonstrates the effectiveness of hyperspectral imaging for differentiating cultivars, growth stages and plant organs of CBD hemp, which is a potentially useful tool for growers and breeders of CBD hemp.

## Introduction

Industrial hemp, or briefly known as hemp, is a crop cultivated for producing a wide range of industrial and consumer products (Renée, 2018). Hemp belongs to the same plant species (*Cannabis sativa* L.) as marijuana that is mainly used recreationally for its intoxicating properties. In the United States, hemp is legally defined as *Cannabis sativa* L. that contains no more than 0.3% total tetrahydrocannabinol (THC), the compound that is responsible for getting a person high and more abundant in marijuana. Because of its association with marijuana, commercial production of hemp in the United States has been long restricted until the passage of the 2018 Farm Bill (Schluttenhofer and Yuan, 2019). As of 2021, all the states in the United States have legalized hemp production for commercial or research purposes. There are three main types of hemp that are grown for different markets, i.e., fiber, oilseed, and cannabidiol (CBD) (Cherney and Small, 2016; Adesina et al., 2020), among which CBD demand is currently the driving force for hemp growth (Carpenter and Peroutek, 2019). While the medicinal uses of CBD are still being researched, market opportunities for CBD hemp are expected to be significant, with CBD sales in the United States projected to reach $23.7 billion by 2023 (Brightfield Group, 2019).

Due to the potential of CBD hemp as an economically viable crop, many farmers are turning to hemp as an alternative crop to fit into their current production system and utilize established farm infrastructure. In a recent survey conducted among North Carolina organic farmers, 85% of the growers expressed interest in growing hemp on their farms and the vast majority intended to grow hemp primarily for CBD (Dingha et al., 2019). As an emerging cash crop, many uncertainties surround producing hemp profitably (Adesina et al., 2020), such as cultivar selection, transplanting dates, planting densities, fertilization, pest management, and harvest dates. Confounding these uncertainties is the federal regulatory limit of THC. Production of hemp with THC levels above 0.3% in the United States can mean the destruction of hundreds of acres and loss of thousands of dollars (USDA-AMS, 2021), which could have been avoided through proper cultivar/variety selection and improved production practices. Hence there is a practical need to identify and discriminate hemp phenotypes and cultivars to facilitate crop management as well as serving forensic purposes. The growth stage of hemp at harvest time, in addition to genetics and environmental factors of seed stocks (Campbell et al., 2019; Glivar et al., 2020), is also an important factor influencing chemical profiles (e.g., THC and CBD) of the plant (De Backer et al., 2012; Stack et al., 2021). It is thus also important to determine growth stages and establish harvest timing recommendations to maximize CBD contents in hemp.

Hemp cultivars and growth stages can be determined by agronomic experts who visually inspect morphological characteristics (e.g., shape, color, and texture) of the plant organs (e.g., leaves and flowers). Visual inspection is affected by inconsistency and variability associated with the perception of inspectors, which is further complicated by significant biological variations within and among hemp cultivars. Some hemp cultivars may not be visually distinct and readily differentiated from each other. Thus, analytical methods, such as gas/liquid chromatography and mass spectrometry (Capriotti et al., 2021), have been proposed for differentiating hemp cultivars based on the chemical fingerprints of the plants (Jin et al., 2017; Wang et al., 2018; Dong et al., 2019). Although accurate and reliable, these methods are slow, costly, require sample preparation and destructive wet-chemistry procedures, and thus are not suitable for rapid, on-site testing applications. Therefore, it would be beneficial if a rapid, non-destructive, and objective method is developed for the differentiation of hemp cultivars as well as growth stages.

Optical sensing technology, which interrogates biological materials non-destructively, is considered an attractive means for addressing the shortcomings of human inspection and analytical testing. Numerous studies have been conducted on using spectroscopic techniques for cultivar/variety differentiation of plants and agricultural products (Cozzolino et al., 2003; Luo et al., 2011; Lu et al., 2014). Recently, Sanchez et al. (2020) used Raman spectroscopy for differentiating hemp, cannabis, and CBD-rich hemp with 100% accuracy. Raman measurements, however, require direct contact of samples with the spectrometer to obtain high-quality signals (Sanchez et al., 2020). Duchateau et al. (2020) used near-infrared spectroscopy for discriminating legal and illegal hemp, defined by a cut-off concentration of 0.2% THC in European Union countries, obtaining classification accuracies of 91–95%. Crushing dried hemp plants was required prior to the spectroscopic measurements (Duchateau et al., 2020). Cirrincione et al. (2021) reported on using attenuated total reflectance infrared spectroscopy for the discrimination between fiber-type and drug-type cannabis samples. Spectroscopic sensing, however, only measures small portions of plant tissues and often requires sample treatments (e.g., drying and grinding) (Duchateau et al., 2020) and direct contact between samples and the detector (Sanchez et al., 2020).

Hyperspectral imaging is a power modality for measuring spectral and spatial information of samples simultaneously (Lu et al., 2020). Compared to spectroscopic techniques that are used for point measurements, hyperspectral imaging is advantageous in delivering reliable and comprehensive analysis of characteristics or properties of plant materials with minimal sample preparation, requiring no sample contact, and thus is potentially more suitable for high-throughput, on-site testing. Pereira et al. (2020) investigated hyperspectral imaging for identifying hemp leaves under natural conditions, achieving sensitivity, and specificity values of 89.45% and 97.60%, respectively. A similar study was conducted by Holmes et al. (2020) on classifying flowers, stems and leaves of hemp using hyperspectral imaging. So far, to the best of our knowledge, no research has been carried out on using hyperspectral imaging for classifying for cultivars and growth stages of CBD hemp.

Given the limitations of existing methods using visual assessment and chemical analysis for phenotyping and characterization of hemp plant materials, the objective of this research is therefore to present a proof-of-concept validation of a novel hyperspectral imaging-based approach for non-destructive, fast, and objective differentiation of cultivars, growth stages and plant organs (i.e., leaves and flowers) of CBD hemp. Specifically, in this research we acquired hyperspectral images from freshly harvested leaf and flower materials of five cultivars of CBD hemp at five growth stages using a benchtop hyperspectral reflectance imaging system, developed an image processing pipeline for segmenting the plant parts from background and extracting spectra from sample segments, performed exploratory analysis of spectral features of hemp samples, and built classification models to differentiate the cultivars, growth stages, and plant parts. This study demonstrates the efficacy of hyperspectral imaging technology as a tool to differentiate cultivars, growth stages and plant parts of CBD hemp, which will be beneficial for hemp cultivation and breeding programs.

## Materials and Methods

### Hemp Samples

**FIGURE 1:**
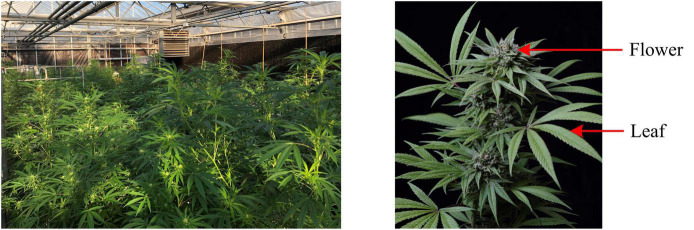
Hemp plants grown in a greenhouse **(left)**, and flower and leaf for sampling **(right)**.

Five CBD hemp cultivars were used in this study, including Cherry Wine (CW), BaOx (BX), First Light 58 (FL58), First Light 70 (FL70), and TJ’s (TJ). These cultivars were chosen as they were used in a complimentary field trial to determine the optimum harvest date, and particularly BX and CW represent the majority of CBD hemp cultivars planted in North Carolina. The hemp plants were grown in a greenhouse, as shown in Figure 1 (left), at the NC State University Horticulture Field Laboratory (Raleigh, NC, United States). The trial was arranged in a complete randomized design containing four replicates. A total of 20 plants (5 harvest dates × 4 replicates) per cultivar were randomly placed on greenhouse benches (total 100 plants for five cultivars).

Hemp harvests took place during September to November of 2020, at 2, 4, 6, 8, and 10 weeks after flower initiation, corresponding to five plant growth stages. At the time of harvest or growth stage, four plants were randomly chosen per cultivar row, corresponding to four replications, and both leaves and flowers, as shown in Figure 1 (right), were sampled for the differentiation of hemp cultivars and growing stages. For each plant, 4 leaves were sampled from its main apical meristem, and 4–6 flowers were sampled depending on the size and number of flowers on the plant. The details of sample numbers are summarized in Table 1. The freshly harvested samples were immediately scanned by a hyperspectral imaging system as described below.

**TABLE 1:** Sample numbers for five hemp cultivars at different growth stages (sampling dates).

Sampling date	Cherry Wine		BaOx		First light 58		First light 70		TJ’s	
	Leaf	Flower	Leaf	Flower	Leaf	Flower	Leaf	Flower	Leaf	Flower
09/24/2020	16	24	16	22	16	16	16	16	16	16
10/08/2020	16	24	16	21	16	17	16	16	16	16
10/22/2020	16	24	16	21	16	16	16	16	16	16
11/05/2020	16	24	16	22	16	16	16	16	16	16
11/19/2020	16	24	16	21	16	16	16	16	16	16

### Hyperspectral Image Acquisition

**FIGURE 2:**
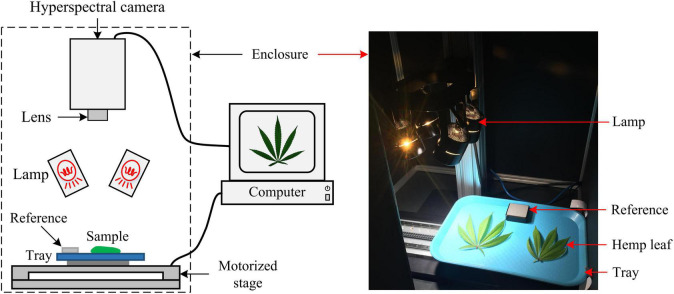
Schematic **(left)** and photograph **(right)** of a hyperspectral imaging system for acquiring images from hemp samples.

A portable, benchtop hyperspectral reflectance imaging system (Figure 2) under controlled lighting was assembled for acquiring images from hemp samples. The system mainly consisted of a line-scan hyperspectral camera (Pika XC2, Resonon Inc., Bozeman, MT, United States), attached with a focusing lens (Xenoplan 1.4/17, Schneider Kreuznach, Bad Kreuznach, Germany), a four-fixture, 140-W halogen lamp assembly (symmetrically oriented with respect to the camera) for providing illumination over samples, a motorized stage (Resonon Inc., Bozeman, MT, United States) and a Spectralon reference target (SRT-20-020, Labsphere, Inc., North Sutton, NH, United States) with nominal reflectance of 20%. Synchronized with the camera, the stage moved a flat sample-holding tray (at a speed of 1 cm/s) for hyperspectral line scanning. The reference, which was placed on the tray and scanned along with samples, as shown in Figure 2 (right), was used for standardizing the spectral responses of the camera. The imaging system was operated in an enclosed chamber to prevent interference from ambient light.

Image acquisitions were conducted on five harvest occasions as indicated above. The hemp leaves and flowers were imaged separately for individual plants. The software SpectrononPro (Resonon Inc., Bozeman, MT, United States) was used for controlling the camera and motorized stage during imaging. The acquired hyperspectral datacube consisted of 462 wavelengths over a wavelength range of 400–1000 nm (at a spectra resolution of 1.3 nm), and spatially each scanning line consisted of 1,600 pixels (at a spatial resolution about 0.5 mm^2^ per pixel for hemp samples), and the number of scanning lines per datacube depended on the actual scanning duration.

### Image Processing

**FIGURE 3:**
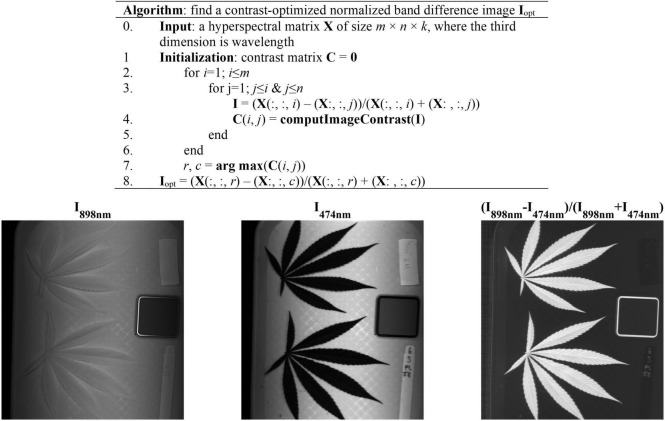
**(Top)** Algorithmic procedures of finding a contrast-optimized, normalized band difference image from a hyperspectral datacube. **(Bottom)** The images at 898 nm and 474 nm and the corresponding normalized band difference image that exhibits the optimal contrast.

The acquired hyperspectral images were processed to segment the reference and hemp samples from the background. Thresholding is a simple and effective technique for image segmentation, provided that the image histogram has well-defined modes corresponding to regions of interests. While a flat, uniformly colored tray (Figure 3) was used as the background for hemp imaging, there was still noticeable illumination unevenness in acquired images (Figure 3), restricting using a global threshold for object segmentation. To facilitate the segmentation, a robust algorithm was developed by obtaining a contrast-optimized, normalized band difference (NBD) image, followed by applying an INTERMODE thresholding technique (Glasbey, 1993; Lu and Lu, 2017). NBD is calculated as vegetation indices in hyperspectral sensing to improve feature discrimination; it can be defined as in a general form (Ferwerda et al., 2005). ***I*** = (***R***_@**λ1**_**−*****R***_@**λ2**_**)/(*****R***_@**λ1**_ **+** ***R***_@**λ2**_**)**, where, ***R***_@__λ1_ and ***R***_@__λ1_ denote the reflectance images at wavelengths λ1 and λ2 (λ1 > λ2), respectively.

In this study, the best wavelength pair was determined, as illustrated in Figure 3, by calculating NBD images for all waveband pairs and choosing the one at which the maximum image contrast is obtained (Lu et al., 2021). The image contrast was defined as the ratio of among-class (plant pixels vs. non-plant pixels) variance to the total variance of an image, following the principle of the Otsu’s thresholding (Otsu, 1979). As such two wavelengths 898 nm and 474 nm, in near-infrared and blue regions, respectively, were identified for calculating NBD images. It is noted that the algorithm was applied to a single hyperspectral image and the identified wavelength pair was then generalized to all other images. As shown in Figure 3, the NDB image is highly contrasted between hemp samples and the background.

**FIGURE 4:**
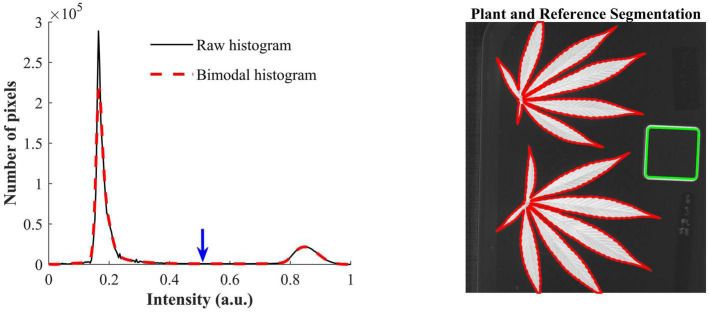
**(Left)** Raw histogram and bimodal histogram of a contrast-optimized normalized band difference image (Figure 3). The blue downward arrow indicates the optimal threshold that corresponds to the mean position of the two peaks of the bimodal histogram that is obtained by iteratively smoothing the raw histogram until it is bimodal. **(Right)** Segmentation of hemp leaves and a reference using the optimal threshold.

The contrast-optimized NBD images enable the segmentation of hemp samples and reference by global thresholding. The histogram of the NBD image, as showed in Figure 4 (left), has two distinct peaks, and the one at the lower end of the histogram corresponds to the background and the other corresponds to hemp samples and the edge of the reference. The INTERMODE thresholding technique finds the optimal threshold by taking the average of the two peaks of a bimodal histogram (Glasbey, 1993; Lu and Lu, 2017). Since the raw histogram might not be ideally bimodal, it was subjected to average smoothing iteratively using a three-point window, before determining the optimal threshold, until the smoothed histogram became bimodal. The thresholding was then followed by routine morphological operations to refine the initial segmentation. Figure 4 (right) shows an example of the segmented hemp leaves and reference.

Furthermore, given a hyperspectral datacube for each scan, mean spectra were extracted for the reference and individual hemp leaves/flower samples, respectively, by averaging the spectra of all the pixels in the corresponding region of interest. Thereafter, ratio spectra were obtained by dividing the spectra of hemp samples by the spectrum of the reference in the same scan, to standardize the spectral responses of the camera, and used for building discriminative models as described below. While morphological or texture features can also be extracted and fused with the mean spectra for modeling tasks, only the latter were used for the modeling tasks and found adequate for yielding high classification accuracies.

### Model Development

**FIGURE 5:**
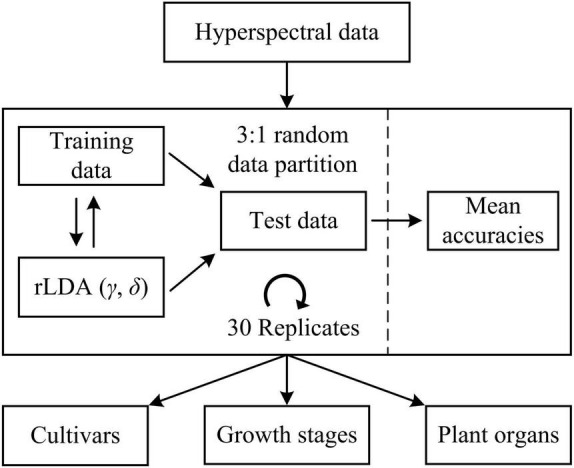
Discriminative modeling of hyperspectral data using regularized linear discriminant analysis (rLDA) for classifying hemp cultivars, growth stages, and plant organs (leaves vs. flowers). For rLDA, two hyperparameters γ and δ are optimized through Bayesian optimization based on 10-fold cross validations during model training.

Machine learning models were developed to differentiate five hemp cultivars, five growth stages (corresponding to five sampling dates) and two plant organs (i.e., leaves and flowers), respectively. For cultivar differentiation, the models were built using the spectra of hemp leaves and flowers, respectively, as well as using the ensemble of hemp leaf and flower samples, at each growth stage. Furthermore, the ensemble of the samples from different stages is also examined for model development. For growth stage differentiation, similarly, classification models were built using hemp leaves and flowers, respectively, for each cultivar. Moreover, models were built for discriminating hemp leaves and flowers for each cultivar at each growth stage. In each modeling scenario, the spectral dataset was randomly partitioned into training and test sets according to a ratio of 3 to 1 (Figure 5), for model training and testing, respectively, and wavelength-wise data normalization was performed so that the reflectance values at each wavelength had a zero mean and a unit variance.

Regularized linear discriminant (rLDA) proposed by Guo et al. (2007) is an extension to the classic LDA specifically for solving classification problems with high-dimensional data. By regularizing the covariance matrix and thresholding (shrinking) the linear coefficients, rLDA is sufficiently robust for modeling high-dimensional data and also very competitive to other far more computation-expensive classifiers such as support vector machine (Guo et al., 2007). Hence, rLDA was chosen for the modeling tasks in this study. There are two tunable hyperparameters in rLDA, i.e., the regularization parameter γ (0 < γ < 1) and threshold (or shrinkage) parameter δ (δ > 0). When there are more predictors (variables) than samples, which typically holds true for high-dimensional data, the optimal value of γ is shifted toward 0 (Guo et al., 2007); a higher value of δ implies fewer variables incorporated into the model, which has the effect of variable selection for modeling. Here the two hyperparameters were determined through Bayesian optimization (Snoek et al., 2012) in the context of 10-fold cross validations on the training data, over a range of [0, 0.01] and [1e-3, 1e3] for γ and δ, respectively. The search ranges were chosen based on preliminary testing. Because of the randomness of the spectral data partition, it would be desirable to repeat the modeling procedures multiple times with random dataset partition for obtaining a reliable estimate of model performance. In this study, a repeated holdout validation strategy (also referred to as Monte Carlo cross validation) was performed to avoid potential pitfalls of single data partition (Raschka, 2018). Given the efficiency of rLDA, a relative high number of 30 modeling replications were conducted (Figure 5), and the resultant mean value of the classification accuracies (the percentages of the number of correctly classified samples of the total sample number) on the test data was computed for model evaluation. Further, statistical comparisons were conducted on the mean classification accuracies among different models using Fisher’s least significant procedure at the 5% significance level.

All the analyses for image preprocessing, feature extraction and model development were performed in Matlab R2020b (The Mathworks, Inc., Natick, MA, United States).

## Results and Discussion

### Exploratory Analysis

**FIGURE 6:**
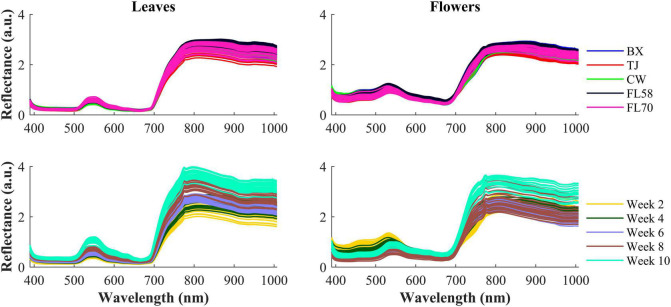
**(Top)** Mean spectra of five cultivars (i.e., BX, TJ, CW, FL58, and FL70) of hemp flowers and leaves harvested 4 weeks after flower initiation and **(Bottom)** mean spectra of the cultivar Cherry Wine harvested at all the five growth stages. The reflectance (a.u.) is a relative quantity obtained by diving a sample spectrum by that of the standard reference (section “Hyperspectral Image Acquisition”) with nominal reflectance of 20%.

Figure 6 shows the spectra of hemp samples of the five different cultivars harvested 4 weeks after flower initiation and the spectra of one cultivar at all the growth stages (2–10 weeks after flower initiation). Like other green plants, the spectra of hemp leaves and flowers are characterized by low reflectance in the visible range due to absorption of plant pigments, and reflectance rising rapidly at wavelengths around 700 nm and plateauing in the NIR region, due to reduced absorption and increased scattering of plant tissues in the region (Horler et al., 1983). The major reflectance valley (i.e., the absorption peak) occurring around 670 nm is attributed to the absorption of chlorophylls. Large spectra variations are observed in the visible (450–650 nm around the green band) and NIR regions, among the samples harvested at different plant growth stages. These variations are associated with the dynamics in the chemical profiles (e.g., pigments and water) of plant organs as the plant matures. It seems more apparent that spectral reflectance of hemp leaves increased with the plant age, which is probably due to leaf senescence-induced degradation of chlorophylls (Merzlyak et al., 1999). Water loss accompanying senescence of plant tissues also contributes to increased reflectance in the NIR range (Hunt and Rock, 1989), which may explain high NIR reflectance of hemp flowers in the last scan.

**FIGURE 7:**
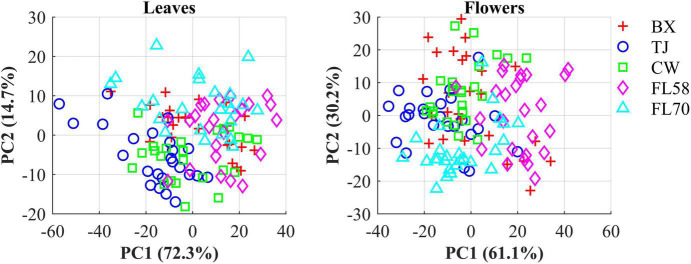
Scatter plots in the space spanned by the first two principal components (PCs) for five cultivars of hemp flowers and leaves 4 weeks after flower initiation (Figure 6). The percentage value in parentheses indicates the variance portion explained by the corresponding PC.

**FIGURE 8:**
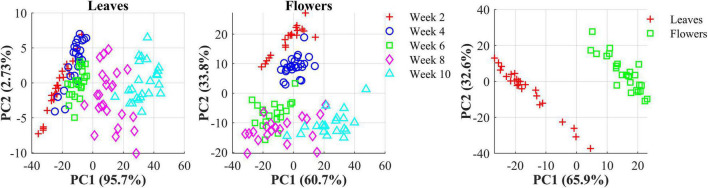
**(Left)** Scatter plots in the space spanned by the first two principal components (PCs) for hemp sample at five growth stages (2–10 weeks after flower initiation). **(Right)** Scatter plots of the first two PCs for hemp flowers and leaves harvested week 4 after flower initiation. The percentage value in parentheses indicates the variance portion explained by the corresponding PC.

The hemp samples cannot be directly distinguished for the five cultivars from the spectral profiles, because of strong overlapping (Figure 6 top); whereas there are more noticeable differences in the spectral profiles among the five growth stages (Figure 6 bottom), and between hemp leaves and flowers. To visualize the distribution of hemp samples of different cultivars, principal component analysis (PCA) was performed on the spectral data. Figure 7 shows an example of the scatter plots for the hemp samples shown in Figure 6. The first two principal components (PCs) account for 87.0% and 91.3% of the total variance of the spectral data of leaf and flower samples, respectively. The scatter plots allow visualizing the unsupervised separation of samples of different classes. Clearly, the samples of five cultivars do not form distinct, well-separated clusters in the PC space, which is also true in the plots by the top three PCs that explain over 96% of the total variance (3D scatter plots are not presented). Similar findings are also observed for the samples harvested at other growth stages. In contrast, the hemp samples among the different growth stages, and especially between plant organs (leaves and flowers) form better-resolved separated groups in the PC space as shown in Figure 8. Despite the qualitative analysis, this result suggests that hemp cultivars would not be readily discriminated though unsupervised analysis, highlighting the need for potent supervised classification techniques to distinguish hemp cultivars, and that high accuracies would be achieved in classifying the growth stages and plant organs.

### Classification

The machine learning models based on rLDA were first developed for differentiating the hemp cultivars using the spectral data of leaf and flower samples separately as well as their combination, at each growth stage (harvest dates). In each scenario, discriminative models were built and tested over 30 replications with random dataset partition for each replication, and the mean overall classification accuracy on test data was calculated and used as the metric of classification performance.

**FIGURE 9:**
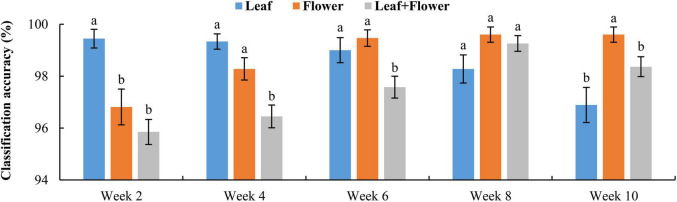
Classification accuracies in differentiating hemp cultivars based on the samples at each growth stage. The classification accuracy is obtained by averaging the accuracies in 30 modeling replicates, and the error bar indicates the corresponding to the (positive/negative) standard error. At each growth stage, the two accuracies with different letters are statistically different at the 5% significance level.

Figure 9 shows the classification accuracy, with statistical comparisons made between the accuracies at each growth stage. Although the PCA of leaf and flower spectra could not reveal a good separation among different hemp cultivars, the rLDA models based on the leaf or flower samples achieved high classification accuracies ranging 96.8% to 99.6%, with standard errors of less than 1%. The classification accuracies vary with plant organ and growth stage. The leaf samples yielded a significantly (+2.6%) better accuracy than that obtained by the flowers at the first growth stage, but a significantly (−2.7%) lower accuracy at the last stage. At the three intermediate stages, the accuracies by the leaves and flowers were similar. It is interesting to note that the accuracy obtained by the leaf samples exhibited a decreasing trend with growth stage, as opposed to an increasing trend for the accuracy by the flowers. The reason underlying this phenomenon has not been fully understood. At week 2, the earliest harvest stage (2 weeks after flower initiation), the flower buds were tiny (3–5 mm) and sticky, which could cause sampling errors. The chemical components (e.g., cannabinoids and cellulose) that are found to be indicative of hemp cultivars **(Sanchez et al., 2020)** may have low concentrations at this stage, which remains to be validated by a further study on chemical analysis of hemp samples. At later growth stages (8–10 weeks after flower initiation), a few hemp plants had minor spider mite (*Tetranychus urticae*) infestation on the leaves, which could also confound the discrimination of hemp cultivars.

**FIGURE 10:**
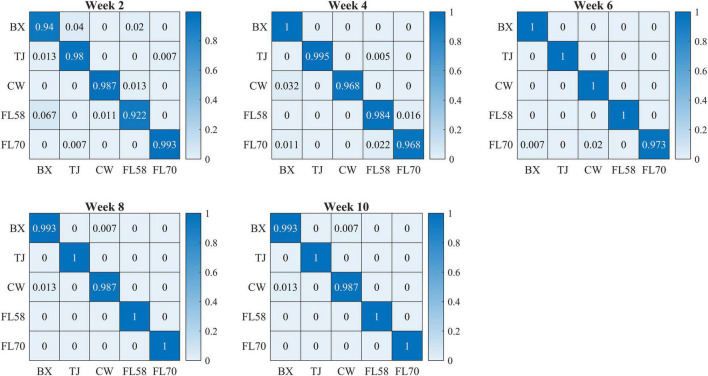
Confusion matrices (rows and columns correspond to true and predicted labels, respectively) for hemp cultivar classification based on the hyperspectral data of flower samples. Each confusion matrix is obtained by pooling and row-wise normalizing classification results over 30 modeling replications.

Figure 10 shows confusion matrices for cultivar classification based on the hyperspectral data of flower samples. Each of the matrices is obtained by pooling and row-wise normalizing the classification results on test data for 30 modeling replications. The overall classification accuracies are similar among hemp cultivars, and there is no consistent pattern of the most or least correctly classified cultivars over the five growth stages. Similar findings were also observed for models based on the data of hemp leaves and the ensemble of flower and leaf samples (confusion matrices not presented). Compared to the results attained using the leaf and flower samples separately, the combined data of yielded statistically diminished or similar accuracy with the lowest and highest values of 95.8% and 99.3% at week 2 and 8, respectively (Figure 9).

**FIGURE 11:**
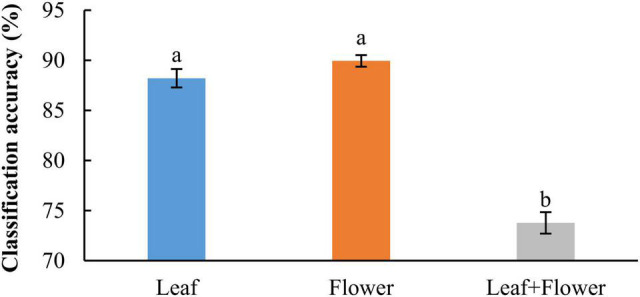
Classification accuracies in differentiating hemp cultivars by pooling samples at all the growth stages. The classification accuracy is obtained by averaging the accuracies in 30 modeling replicates, and the error bar indicates the corresponding to the (positive/negative) standard error. The two accuracies with different letters are statistically different at the 5% significance level.

**FIGURE 12:**
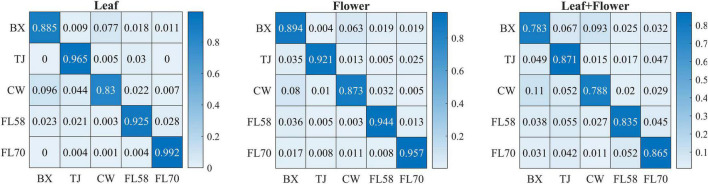
Confusion matrices for hemp cultivar classification using the samples from all the growth stages. Each confusion matrix is obtained by pooling and row-wise normalizing classification results over 30 modeling replications.

In addition to modeling the samples at individual growth stages, the samples collected from different growth stages were also pooled together to build models for cultivar classification. Here, three types of models were built by pooling all the leaf samples, the flower samples and their combination at the five growth stages, resulting in the accuracies of 91.9%, 91.8%, and 82.8%, respectively, as shown in Figure 11. The combination of leaf and flower samples led to a significantly lower accuracy than modeling them separately. Compared to the results of models for individual growth stages (Figure 9), the accuracies obtained from pooling the samples across growth stages resulted in a marked accuracy reduction of 5.03% to 16.4%. This is likely because of the added variations or complexities (e.g., in flower morphology and chemical constituents, and pest infections in leaves) that could not be well modeled by the rLDA classifier using existing datasets. Upon examination of the corresponding confusion matrices (Figure 12), the misclassification between the cultivars BaOx (BX) and Cherry Wine (CW) contributed the most to the overall accuracy deterioration, while comparable, noticeably higher accuracies were obtained for the other three cultivars. Although modeling the leaf or flower samples alone at similar growth stages led to better accuracy in classifying hemp cultivars, it would be desirable to have models that are robust to variations associated with plant organs and growth stages. Hence it is worthy of further investigations to exploit more advanced pattern classification algorithms, on a larger, more diverse set of hemp samples, to improve the accuracy of cultivar classification regardless of growth stages or plant organs.

Overall, these classification results demonstrate that hyperspectral imaging coupled with supervised modeling is a viable means for differentiating hemp cultivars with high accuracy, and that the growth stage and plant organ need to be factored in developing cultivar classification models.

**FIGURE 13:**
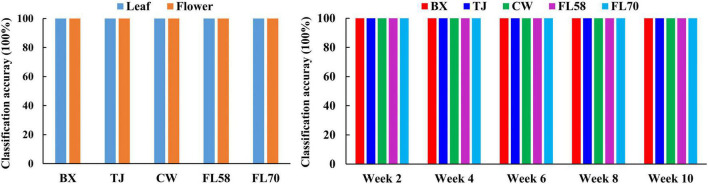
Classification accuracies in differentiating plant growth stages **(left)** for each hemp cultivar (i.e., BX, TJ, CW, FL58, and FL70) and plant organs **(right)** at each growth stage. 100% accuracy is obtained in all the modeling scenarios with zero standard deviation for 30 replications.

Furthermore, rLDA models were built for discriminating the five growth stages and plant organs (leaf and flower) for each of the five hemp cultivars. The classification accuracies of 100% with zero standard error in 30 modeling replications (Figure 13) were obtained in all the scenarios. The superior results are not unexpected given the clear separation of different categories observed in the PCA space (Figure 8). The results are also in good agreement with the findings in literature. Borille et al. (2017) achieved 100% accuracy in discriminating three growth stages of *Cannabis sativa* using NIRS combined with support vector machine. Holmes et al. (2020) applied hyperspectral imaging in 900–1700 nm for discriminating flowers, stems and leaves of *Cannabis sativa* and achieved near 100% precision based on decision tree modeling. All these findings conceivably verify the prowess of hyperspectral imaging for accurately discriminating plant growth stages and organs (leaf and flower). Moreover, the perfect classification of growth stages can be potentially beneficial for improving the classification of hemp cultivars at varying growth stages by deploying cascade classifiers for classifying both hemp growth stages and cultivars.

### Discussion

It is important to point out potential areas for further improvements. Although the standard reference (2” × 2” in size) was scanned along with hemp samples for spectral correction, it was not still sufficient for accounting for the spatial non-uniformity of illumination over the scanning line. It is more desirable to use a larger reference for imaging so that the spectral correction of samples can be performed at a pixel level along the scanning line. An alternative solution is to improve the lighting design to provide uniform illumination over samples. Using two line-light illuminators positioned symmetrically to the camera axis (Ariana and Lu, 2010), instead of the four-lamp setup in this study (Figure 2), may improve the illumination uniformity. This research and other previous studies on hyperspectral imaging for cannabis plants or hemp (Duchateau et al., 2020; Holmes et al., 2020; Pereira et al., 2020) did not consider spatial or textural features for modeling. Arguably these features are also useful for plant classification tasks such as cultivar differentiation, since different cultivars of hemp leaves and flowers are likely to have different morphological features, regardless of growth stages, based on which experienced agronomic experts tell apart different crop cultivars. Further research is hence warranted to extract textural features and exploit strategies of fusing them with spectral features for improving the differentiation of hemp cultivars. Meanwhile, wavelength selection or dimension reduction (e.g., PCA) can be conducted to facilitate texture feature extraction.

The present study only conducted hyperspectral scanning for sampled plant organs (e.g., leaves and flowers) in controlled-light settings. For high-throughput testing and a further validation of the hyperspectral imaging approach, further studies are needed to perform *in-situ* scanning of hemp plants under natural light conditions, requiring no sampling of plant parts. Harvesting hemp for maximum CBD yield while avoiding THC exceeding legal thresholds requires quantification of these chemical compounds in plant organs (especially flowers). Investigations are underway to determine the feasibility of using hyperspectral imaging for screening hemp genotypes based on CBD and THC concentrations in plant tissues at different growth stages.

## Conclusion

In this study we propose a new methodology of using hyperspectral imaging for differentiating cultivars, growth stages, and plant organs (leaves and flowers) of CBD hemp. Fresh leaves and flowers of five hemp cultivars, harvested at five growth stages 2–10 weeks after flower initiation, were scanned by a benchtop hyperspectral reflectance imaging system in the wavelength range of 400–1000 nm. An image processing algorithm was developed for segmenting samples from background. The spectral profiles and PC score scatter plots of hemp samples, to a varying degree, revealed the separation among the hemp cultivars, growth stages and plant organs. The rLDA models, using leaf or flower samples at individual growth stages, achieved the classification accuracies of 96.8%-99.6% in the differentiation of hemp cultivars. Pooling leaf and flower samples at all growth stages resulted in deteriorated accuracies compared to modeling samples at individual growth stages. Both growth stages and plant organs need to be factored in model development for hemp cultivar classification. In contrast, in the differentiation of growth stages and plant organs, the rLDA models achieved 100% accuracies consistently. This study shows that hyperspectral imaging can be used for non-destructive and accurate differentiation between hemp cultivars, growth stages and plant organs, and it is a potentially valuable tool for phenotyping, cultivar selection and optimization of harvest timing in CBD hemp production. Extensive research is still needed to develop and deploy hyperspectral imaging technology for field-scale, *in-situ* applications.

## Data Availability Statement

The raw data supporting the conclusions of this article will be made available by the authors, without undue reservation.

## Author Contributions

YL: conceptualization, methodology, data collection, final analysis, writing – original draft preparation, reviewing, and editing. SY: data collection, writing, reviewing, editing, and project administration. EL: data collection, writing, reviewing, and editing. BW: resources, writing, reviewing, and editing. DS: data collection, resources, writing, reviewing, and editing. All authors contributed to the article and approved the submitted version.

## Conflict of Interest

The authors declare that the research was conducted in the absence of any commercial or financial relationships that could be construed as a potential conflict of interest.

## Publisher’s Note

All claims expressed in this article are solely those of the authors and do not necessarily represent those of their affiliated organizations, or those of the publisher, the editors and the reviewers. Any product that may be evaluated in this article, or claim that may be made by its manufacturer, is not guaranteed or endorsed by the publisher.
